# Our Correspondence

**Published:** 1879-01

**Authors:** 


### Our Correspondence.

NAVASOTA, TEXAS, Nov. 11, 1878.

DR. T. S. UP DE GRAFF:

*Dear Sir:*—Thanks for publishing in the October number of the BISTOURY my account of Mr. Tieman, the man who was relieved from the curse, or sentence pronounced on Adam and his offspring in Genesis, 3d chapter and 19th verse. But I have to correct one error and show a fault in your proof-reader for allowing such an egregious blunder to pass as that where the name of Galileo is put in the place of Gallio, who figures in New Testament history in Acts, 18th ch., 12 v., *et seq.*, where Paul was on trial for charges of false teaching by the Jews, before Judge Gallio, or, more properly, the Proconsul of Achaia.

I presume your proof-reader is more familiar with philosophical reading, or astronomical celebrities, than he is with theology, or the sacred Scriptures. I fear he never attended Sunday school, or learned his Catechism well.

Junius Annacus Gallio was Proconsul under Emperor Claudius in the year A. D. 53, and was the brother of the celebrated Roman philosopher, Lucius Annacus *Seneca*, but he was a rollicking fellow, fond of good eating and high living, and in a foolish moment committed suicide, and probably some "compunctious visitings of conscience" may have helped to impel him to the rash act. Pontius Pilate, wearied with misfortunes and haunted with the memories of the crucifixion of Christ, after his banishment from Rome to Vienna Allobrogum, committed suicide by drowning in Lake Lucerne, where a quadrangular rock, called Mount Pilatus, perpetuates his name.

The proof-readers and printers in the New York *Herald* office have lately gone through a course of severe criticism and trial, many losing their places on account of blunders in quotation, especially in Bible quotations.

We have several Jews residing here, and the operation of circumcision has become almost as familiar as baptism, and Rabbi is nearly as much repeated as Parson. I heard a gentleman one day speaking of such a one having his child circumcised by the *Arab*, and I was afraid to laugh, or correct him. Men are liable to make a *lapsus peunæ*, or a *lapsus linguæ*, but proof-readers have no excuse for changing copy without they are certain they are improving it.

Now I think I am fully justifiable in introducing Judge Gallio when I did, because Mr. Tieman was in court, and was beseeching the Judge and the lawyers to detain him no longer than they could help, but they paid no attention to his plea and were perfectly indifferent to his comfort, or welfare, even if he suffered torture—or death—so Gallio was indifferent about Paul, or his accusers. But Galileo was no lawyer, no judge, and the only crime (?) he was ever accused of was telling the world that our earth revolved on its axis and around the sun, which the Bishops, Cardinals and Pope said, was contrary to Scripture, and he was made to hush up and then cast into prison for it: I feel like putting that proof-reader there too! So, there now.

Yours truly, A. R. K.

Serves him right, Doctor. He "acknowledges the corn," declaring that he was in supreme ignorance of the name of Gallio. He has purchased a new Bible, and declares that you shall not catch him again.

---

ITHACA, N. Y., Dec. 10, 1878.

DR. THAD S. UP DE GRAFF:

*Dear Sir:*—In your lecture last night, for the benefit of the Home, you told us not to wear spectacles unless they were properly fitted. How is that to be done? MRS. M.

We were careful to say that spectacles should always be fitted by a competent oculist, who, with his ophthalmoscope and test-glasses, can determine accurately the character and strength of the lenses required. Spectacles, we repeat, should never be worn, unless so selected. There exist a variety of anomalies of refraction with which the spectacle-dealer is not familiar, therefore, making it impossible for him to fit lenses to all eyes. We are not unfrequently called upon to prescribe for weak and inflamed eyes that have been rendered so by improper spectacles.

The oculist is the proper person to make a prescription for spectacles.

---

HANCOCK, N. Y., Nov. 21, 1878.

DR. UP DE GRAFF:

How can I determine whether a person is blind from *cataract* or from an opaque spot on the cornea. My father, aged 80, has gradually grown blind until now he is unable to find his way. He has had no pain or inflammation, but his pupils look very white. Physicians have called it *cataract* and opacity of the cornea, and I'm sure I cannot decide when doctors disagree. D. M.

Doubtless, your father has *cataract*. If it were opacity of the cornea, he would have had severe inflammation and ulceration of the cornea, before the scar or opacity could form. Place your hand over his eyes for a few minutes, then suddenly remove it and look at his pupils: they will be dilated, and the opaque spot will appear correspondingly enlarged and just the size and shape of the pupil. Opacity of the cornea is not regular in outline, may be over the centre, or to one side of the cornea, while the dark pupil can be seen underneath.

No pain accompanies cataract. Opacity of the cornea is produced by an inflammation that is *always* painful.

---

ITHACA, N. Y., Oct 14, 1878.

DEAR DOCTER:

Thanks for the entertaining and instructive BISTOURY. I think the "Dobson" must be the "Horned Corydalus," or *corydalus cornutus.*

Yours Truly, B. G. W.

---

MIDDLETOWN, N. Y., Oct. 15, 1878.

DR. UP DE GRAFF:

I saw in your October No. of the BISTOURY, an article on the "*Dobson.*" I have used the same thing, which are to be found along the banks of the Delaware, at Port Jervis. The Bass takes right hold of them. They are called "*Hojacks*" by the people in this section, and are valued highly as bait for Bass. I don't know how the "Pickerel" like them, never having tried them but once. *

---

KING'S FERRY, N. Y., Dec. 20, 1878.

DR. UP DE GRAFF:

I have read in the Elmira *Advertiser*, a letter from a Sergt. Potter, complaining of the food and clothing furnished the prisoners in the Elmira prison, during the war.

I have repeatedly conversed with those who had visited the Elmira prison, all of whom spoke of the excellent food, clothing and shelter that were afforded the confederates, while they complained bitterly of the bad treatment of our own soldiers confined in southern prisons.

I made one of the many officers who spent the winter of 1864–5, in Libby Prison, and in no fault finding mood, and with no desire to stir up any bitter feelings between the soldiers of the north and south, I desire to call Serg't. Potter's attention to the bill of fare we had in Libby. Perhaps he will feel more kindly toward the Elmira prison, knowing how we suffered in the prisons of his government.

We had two meals a day in Libby, at 11 a. m., and 3 p. m. They consisted invariably and uninteruptedly of corn bread—a piece two inches square—made from unbolted corn meal; and a half pint of pea soup. I have many times aided in skimming the black bugs from the top. Sometimes we were allowed a piece of fresh meat, of two ounces weight, when they had it to give, then the pea soup was witheld. The rations were so scanty as to produce the most frightful consequences among those that were able to survive it. But of this your readers had sufficient information through the newspapers, at the time. On Christmas day, we had bread alone, of the color and consistency of ginger bread, which we really thought it to be, in consideration of the day, but which proved to have been made from ground sorghum seed.

Our last ration I remember well. It was a very small ham, intended to appease the appetites of fifty officers. When we cut it open, we passed it around on a *spoon.*

Those of the officers or men who were fortunate enough to have money, could buy a few delicacies at the following rates: $2.50 bought a diminutive loaf of bread; $1.00 an onion; a fair sized potato was rated at a dollar, while the same sum was asked for a tablespoonful of rice. Every Sunday, we indulged in the luxury of a "tallow dip." For this we paid $2.50. By the light of this a christian clergyman from New Hampshire used to preach to us. I am sorry I have forgotten his name—but I will right here add my testimony to the christian spirit of forgiveness and unselfish devotion to the welfare of those by whom he was surrounded, during that long dreary winter. He was a noble man.

Believe me, I bear no malice nor the slightest ill-feeling toward our captors, or the authorities who kept us there so long, at death's door. Indeed, I believe they fed us as well as they could—according to their means. I know the soldiers who guarded us, complained most bitterly of their meagre rations. I also know, from personal inspection, that our government gave the confederate officers imprisoned in Hilton Head, S. C., a princely allowance of food, compared with what we were furnished at Libby; and these Hilton Head officers were supposed to be placed on "short allowance" of rations as a matter of retaliation for what we suffered in Libby.

Sometime I may give the readers of the BISTOURY, a letter on the inside life in Libby. Until then, bye bye,

F. A. DUDLEY, M. D.,
Late Surgeon 14th Conn. Inft'y.

